# Mild and Highly Efficient Stereoselective Synthesis of 2,3-Unsaturated Glycopyranosides using La(NO_3_)_3_ · 6H_2_O as a Catalyst: Ferrier Rearrangement

**DOI:** 10.1080/00397910701744170

**Published:** 2008-01-18

**Authors:** N. Suryakiran, S. Malla Reddy, M. Srinivasulu, Y. Venkateswarlu

**Affiliations:** Organic Chemistry Division I, Natural Products Laboratory, Indian Institute of Chemical Technology, Hyderabad, India

**Keywords:** alcohols, allyl TMS, La(NO_3_)_3_ · 6H_2_O, phenols, thiols, 3,4,6-tri-*O*-acetyl-D-glucal

## Abstract

A mild and highly efficient stereoselective reaction of 3,4,6-tri-*O*-acetyl-D-glucal with a variety of nucleophiles, viz. alcohols, phenols, thiols, thiophenols, and allyl trimethyl silane (TMS), in the presence of 5 mol% of lanthanum(III) nitrate hexahydrate under solvent-free conditions yielded the corresponding 2,3-unsaturated glycopyranosides (pseudoglycals) in excellent yields.

## 1 INTRODUCTION

The anomeric *C*-glycolysation is an important transformation for the synthesis of aryl and alkyl 2,3-unsaturated glycopyranosides, as they are very important chiral intermediates in the synthesis of several biologically active natural products,^[^1^]^ uronic acids,^[^2,3^]^ modified carbohydrates,^[^4,5^]^ nucleosides antibiotics,^[^6,7^]^ and oligosaccarides,^[^8–10^]^ and they are also common structural units in many medicinally significant molecules such as antibiotics.^[^11^]^ The direct and straightforward method for the synthesis of this class of compounds is an allylic rearrangement of glycols by the anomeric glycosidation with different nucleophiles in the presence of Lewis acids. This is well known as the Ferrier rearrangement,^[^12^]^ which includes BF3OEt2,^[^12–14^]^ SnCl_4_,^[^15,16^]^ InCl_3_,^[^17^]^ montmorillonite K-10,^[^18^]^ 2,3-dichloro-5,6-dicyano-*p*-benzoqui-none (DDQ),^[^19^]^ N-iodosuccinimide (NIS),^[^20^]^ I_2_,^[^21^]^ FeCl_3_,^[^22^]^ and some metal triflates such as Dy(OTf)_3_^[^23^]^ and Yb(OTf)_3_.^[^24^]^ However, the use of strongly acidic conditions frequently leads to the formation of undesirable side products competing with the main reactions. Thus, a mild and efficient catalyst for the synthesis of 2,3-unsaturated glycopyranosides is highly desirable. In view of current interest in catalytic processes, there is a merit in developing the synthesis of pseudoglycals using an inexpensive, mild, and nonpolluting reagent.

Organic reactions using mild and water-tolerant catalysts received much attention in recent years. They can be conveniently handled and removed from the reaction mixture, making the experimental procedure simple and eco-friendly. Lanthanum(III) nitrate hexahydrate is relatively nontoxic, inexpen-sive, insensitive to air, and used in various organic transformations, such as chemoselective tetrahydropyranylation of primary alcohols,^[^25^]^ chemoselec-tive deprotection of acetonides,^[^26^]^ synthesis of quinazolinones,^[^27^]^ mild and efficient acetylation of phenols and amines,^[^28^]^ synthesis of α-amino nitriles,^[^29^]^ synthesis of benzodiazepines,^[^30^]^ and *N*-*tert*-butoxycarbonylation and *N*-benzyloxycarbonylation of amines.^[^31,32^]^ In studying these transform-ations, it has been observed that the substrates containing other acid labile functional groups, such as TBDMS ethers, some isopropylidene protected diols, and *N*-*tert*-Boc-protected amines, were intact in the presence of La(NO_3_)_3_ · 6H_2_O. In continued efforts for utilizing La(NO_3_)_3_ · 6H_2_O, we found that it is an efficient and mild Lewis acid catalyst for the synthesis of 2,3-unsaturated glycopyranosides.

## 2 RESULTS AND DISCUSSION

**Scheme 1.**
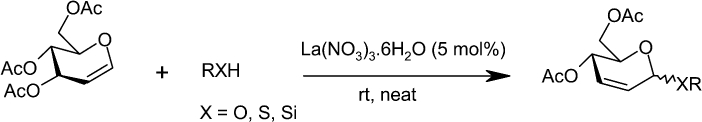


**Scheme 2.**
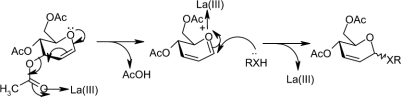
Proposed mechanism

In this article (Scheme 1), we describe a mild and efficient method for the stereoselective synthesis of 2,3-unsaturated glycopyranosides in excellent yields with α-selectivity. This method is very inexpensive, and no special care is required to exclude moisture from the reaction medium. La(NO_3_)_3_ · 6H_2_O is highly oxophilic, forms a labile bond with carbonyl oxygen and initiates the formation of a C−X (X = O, S, Si) bond with nucleophiles (Scheme 2). The reaction of 3,4,6-tri-*O*-acetyl-D-glucal with different nucleophiles in the presence of a catalytic amount of lanthnum(III) nitrate hexahydrate under solvent-free conditions at room temperature proceeds efficiently and smoothly, and the reaction conditions are very mild. No by-products were observed. Furthermore, other functionalities such as *N*-*tert*-butylcarbamates are compatible under reaction conditions (Table 1, entries 5 and 14). This also indicates that *N*-*tert*-Boc-protected amino acids are suitable substrates for this reaction. We first examined the reaction of 3,4,6-tri-*O*-acetyl-D-glucal and homo-allyl alcohol in the presence of La(NO_3_)_3_ · 6H_2_O (5 mol%) under solvent-free conditions (Table 1, entry 4) to give corresponding 2,3-unsaturated glycopyranosides in 94% yield with high α-selectivity (10.5:1). Encouraged by this result, we extended the generality of the reaction with different nucleophiles such as alcohols, phenols, thiols, and allyl TMS at room temperature to give corresponding 2,3-unsaturated glycopyranosides in excellent yields (Table 1). In most of the cases, products were obtained as a mixture of α- and β-anomers, with the α-anomer being favored. The α-to β-ratio was deter-mined on the basis of integration ratios of the anomeric protons in their corresponding ^1^H NMR spectrum.

**Table 1.**
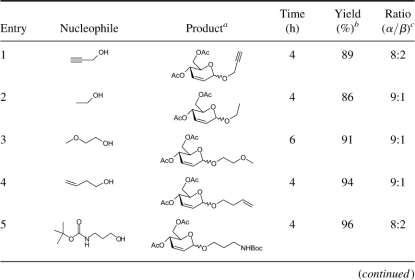

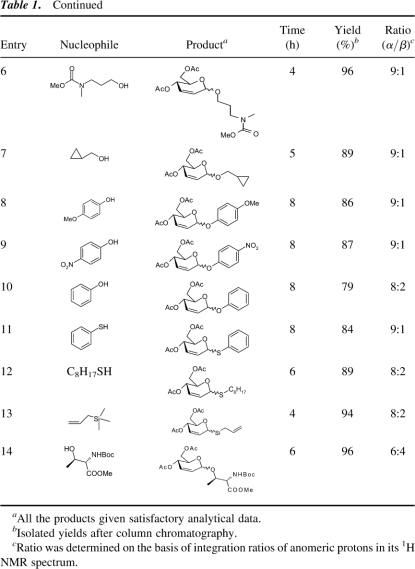
Synthesis of 2,3-unsaturated glycopyranosides with 3,4,6-tri-*O*-acetyl-D-glucal using La(NO_3_)_3_ . 6H_2_O as a mild and efficient catalyst under solvent-free conditions

## 3 CONCLUSION

In conclusion, we have described a mild and efficient method for the synthesis of 2,3-unsaturated glycopyranosides using La(NO_3_)_3_ · 6H_2_O under solvent-free conditions.

## 4 EXPERIMENTAL

### 4.1 Typical Experimental Procedure for the Synthesis of 2,3-Unsaturated Glycopyranosides

La(NO_3_)_3_ · 6H_2_O (5 mol%) was added to a mixture of 3,4,6-tri-*O*-acetyl-D-glucal (1 mmol), alcohols/phenols/thiols/allyl TMS (1.1 mmol), and the reaction mixture was stirred at room temperature under solvent-free conditions for the appropriate time (Table 1). After completion of the reaction as monitored by thin-layer chromatography (TLC), water was added, and extracted into ethyl acetate. The organic layer was dried over anhydrous sodium sulphate and evap-orated under reduced pressure. The crude product was purified over silica gel to yield the corresponding 2,3-unsaturated glycopyranosides.

### 4.2 Spectral Data for Selected Compounds

Entry 3: Solid, mp 44–46°C; [α]_D_^25^ 82.2 (*c* = 2.0, CHCl_3_); ^1^H NMR (CDCl_3_, 200 MHz): δ 2.02 (s, 6H, COCH_3_), 3.30 (s, 3H, OMe), 3.46–3.50 (m, 2H, OCH_2_), 3.55–3.62 (m, 1H, OCH_2_), 3.77–3.92 (m, 1H), 3.97–4.17 (m, 3H), 4.95 (b s, 1H, H-1), 5.20 (dd, 1H, J_3,4_ = 1.20 Hz, J_4,5_ = 9.80 Hz, H-4), 5.77 (m, 2H, H-2 & H-3); IR (KBr) υmax: 3374, 2926, 1746, 1544, 1451 cm^−1^; LCMSD: *m*/*z* 289 (M^+^ + 1). Entry 4: solid, mp 49–52°C; [α]_D_^25^ 80.9 (*c* = 1.2, CHCl_3_); ^1^H NMR (CDCl_3_, 300 MHz): δ 2.07 (s, 6H), 2.35 (dt, 2H, *J* = 1.50, 6.80, 8.30 Hz), 3.51–3.58 (m, 1H), 3.75–3.83 (m, 1H), 3.99–4.01 (m, 1H), 4.14–4.20 (m, 2H), 4.97 (b s, 1H, H-1), 5.02–5.12 (m, 2H), 5.24 (dd, 1H, *J* = 1.60, 10.57 Hz), 5.81 (m, 2H); LCMSD: *m*/*z* 285 (M^+^ + 1).

Entry 5: [α]_D_^25^ 64.0 (*c* = 1.2, CHCl_3_); ^1^H NMR (CDCl_3_, 200 MHz): δ 0.90 (m, 2H), 1.22 (s, 9H), 1.38–1.40 (m, 3H, NH, NCH_2_), 2.09 (s, 3H), 2.10 (s, 3H), 3.52 (m, 1H), 3.71 (ddd, 1H, *J* = 3.34, 6.69, 10.0 Hz), 3.89 (m, 1H), 4.30 (dd, 1H, *J* = 2.50, 12.54 Hz), 4.44 (dd, 2H, *J* = 4.18, 12.54 Hz), 4.65 (dd, 1H, *J* = 2.5, 5.86 Hz), 5.28 (dt, 1H, *J* = 1.67, 2.41, 4.10 Hz), 6.37 (dd, 1H, *J* = 1.67, 6.69 Hz); LCMSD: *m*/*z* 388 (M^+^ + 1).

Entry 6: Solid, mp 50–52°C; [α]_D_^25^ 96.4 (*c* = 1.2, CHCl_3_); ^1^H NMR (CDCl_3_, 200 MHz): δ 1.78 (m, 2H), 2.00 (s, 6H), 2.86 (s, 3H, NMe), 3.2–3.50 (m, 3H), 3.60 (s, 3H, COOMe) 3.71–3.88 (m, 1H), 3.94–4.16 (m, 3H), 4.90 (b s, 1H, H-1), 5.20 (dd, 1H, *J* = 1.20, 9.60 Hz), 5.75 (m, 2H); IR (KBr) υmax: 3414, 2927, 1742, 1664, 1660, 1542, 1456, 1444, 726 cm^−1^; FAB mass: *m*/*z* 360 (M^+^ + 1).
